# Economic evaluation of the Good School Toolkit: an intervention for reducing violence in primary schools in Uganda

**DOI:** 10.1136/bmjgh-2017-000526

**Published:** 2018-04-17

**Authors:** Giulia Greco, Louise Knight, Willington Ssekadde, Sophie Namy, Dipak Naker, Karen Devries

**Affiliations:** 1 Department of Global Health and Development, London School of Hygiene and Tropical Medicine, London, UK; 2 School of Economics, Makerere University, Kampala, Uganda; 3 MRC/UVRI Uganda Research Unit on AIDS, Uganda; 4 World Education International/Bantwana, Kampala, Uganda; 5 Raising Voices, Kampala, Uganda

**Keywords:** economic evaluation, cost-effectiveness analysis, violence against children, violence in schools, primary education, Uganda

## Abstract

**Introduction:**

This paper presents the cost and cost-effectiveness of the Good School Toolkit (GST), a programme aimed at reducing physical violence perpetrated by school staff to students in Uganda.

**Methods:**

The effectiveness of the Toolkit was tested with a cluster randomised controlled trial in 42 primary schools in Luwero District, Uganda. A full economic costing evaluation and cost-effectiveness analysis were conducted alongside the trial. Both financial and economic costs were collected retrospectively from the provider’s perspective to estimate total and unit costs.

**Results:**

The total cost of setting up and running the Toolkit over the 18-month trial period is estimated at US$397 233, excluding process monitor (M&E) activities. The cost to run the intervention is US$7429 per school annually, or US$15 per primary school pupil annually, in the trial intervention schools. It is estimated that the intervention has averted 1620 cases of past-week physical violence during the 18-month implementation period. The total cost per case of violence averted is US$244, and the annual implementation cost is US$96 per case averted during the trial.

**Conclusions:**

The GST is a cost-effective intervention for reducing violence against pupils in primary schools in Uganda. It compares favourably against other violence reduction interventions in the region.

**Key questions**

**What is already known?**

- The literature on ‘what works’ to prevent violence against children is growing but still limited.
- There are no studies that test the effectiveness and cost-effectiveness of interventions to reduce and prevent violence from school staff to students.

**What are the new findings?**

- The Good School Toolkit (GST) represents a locally developed, behavioural intervention which requires comparatively little resources at the school level to significantly reduce physical violence by teachers against students.
- The cost per case of violence averted compares favourably with other violence prevention and reduction interventions.

**What do the new findings imply?**

- It is of critical importance to identify and implement sustainable and cost-effective interventions for the prevention of violence against children, such as the GST, especially in resource-poor settings.

## Background

Violence against children has damaging and long-lasting effects. Children who have been victims of violence have an increased risk of developing mental health problems,^1^ they perform worse in school,^2^ and are more likely to be victims or perpetrators of intimate partner violence (IPC) later in life.^3 4^

Physical violence against children in school is widespread in East Africa. In Kenya, 40% of students aged 13–17 years reported being punched, kicked or whipped by a teacher in the past week; in Tanzania, 50% reported physical violence from a teacher when they were under 18 years of age.^5 6^ In Uganda, a survey in Luwero District revealed that violence in schools is a near universal experience for students: 92% of children aged 11–14 have ever experienced physical violence inflicted from school staff, and 52% during the past week.^7^ Eight per cent of lifetime physical violence is severe, including burning, choking and stabbing or being severely beaten up. Four per cent of all students have sought medical attention because of an injury from violence caused by a school staff member.

The global costs and economic impacts of physical, psychological and sexual violence against children have been estimated at a staggering US$7 trillion, equivalent to 8% of global gross domestic product.^8^ There is a strong case for investing in violence prevention programmes to avoid the negative physical, emotional and economic consequences that acts of violence have on children, their families, their peers and their communities.^8^ However, the literature on ‘what works’ to prevent violence against children is growing but still limited. To our knowledge, there are no studies that test the effectiveness and cost-effectiveness of interventions to reduce and prevent violence from school staff to students.

This paper presents the cost and cost-effectiveness analysis of developing and implementing an intervention aimed at reducing violence against children: the Good School Toolkit (GST). The intervention was tested with a cluster randomised controlled trial for its effectiveness in reducing physical violence by school staff in 42 primary schools in Luwero District, Uganda.^9 10^

## Methods

### The Good School Toolkit

The Good School Toolkit is a complex behavioural intervention which aims to change the operational culture in schools. It was developed by Raising Voices, a Ugandan non-governmental organisation (NGO), over a period of 6 years (2007–2012) in partnership with pilot schools in Kampala. It is freely available at http://raisingvoices.org/good-school/.

The Good School Toolkit builds on the idea that the operational culture of the school—the way in which stakeholders experience, behave and feel at their school (Cohen 2006)—impacts the level of violence children are experiencing in their learning environment. As such, GST adopts an ecological framework and holistically assesses the various layers of influence affecting learners, including individual, interpersonal, community and societal.

The Toolkit itself is a six-step process containing about 60 activities coordinated at the school level by two teacher ‘protagonists’, two student representatives and two school-affiliated community members. These activities are related to fostering a conducive learning environment, mutual respect and empathy, understanding power relationships, using non-violent positive discipline alternatives and improving teaching techniques.^11^

Collectively these six steps are designed to build upon one another based on the transtheoretical model of behaviour change, which helps understand the process an individual goes through when contemplating, preparing for, and acting on and maintaining changes in their behaviour.^12^

The Toolkit contains behavioural change techniques that have been shown to be effective in relation to a range of different behaviours. These include: setting school-wide goals, developing action plans with set dates for deliverables, encouraging empathy by facilitating reflection on experiences of violence, providing positive discipline tools and promoting opportunities to practise newly acquired behavioural skills.

Schools receive support by the Raising Voices team and are encouraged to monitor and assess their progress according to their action plans. They also receive in-school support by teacher protagonists to their peers as they gain new knowledge and skills. Pupils are active participants as they create committees and groups related to different activities. Goal achievements and action plan deliverables are celebrated and rewarded. Different groups (teachers, administration, students and also parents) engage in the activities, thus wider social support is created to ensure stable behavioural changes. The Toolkit materials consist of training and facilitation guides, booklets and posters for the school-based activities.

In each school, activities are led by two teacher and two student protagonists who receive a 3-day training workshop and continuous mentorship and support from Raising Voices staff. Raising Voices staff also provide three support visits per term. The aim of the first visit is to provide support for action plan development, second visit for tracking development, and the third one for technical support. As part of the trial, a dedicated Study Process Monitor (M&E) conducted two visits per term to each intervention school during the implementation period. The first visit included a classroom observation^13^ and school-wide assessment, and the second impromptu visit aimed to include observation of a school-led Toolkit activity planned for that time in schools. The Study Process Monitor collected and filed completed school-led activity sheets and Toolkit termly action plans. Additionally, during the final term of implementation, the Study Process Monitor conducted weekly phone calls to school protagonists to monitor and record details of school-led activities conducted. Summary monitoring reports that described monitoring activities and in-school observations were documented and shared with the Good School programme team termly. Control schools also received the first visit.

### The Good Schools Study

The Good Schools Study (GSS) is a two-arm cluster randomised controlled trial conducted in 42 primary schools in Luwero District, Uganda.^9 10^ Cross-sectional baseline and end line surveys were conducted in schools in June to July 2012 and June to July 2014, respectively. The implementation period lasted 18 months. The primary outcome was past-week physical violence from a school staff member, self-reported by pupils following the International Society for the Prevention of Child Abuse and Neglect Screening Tool-Child Institutional. The secondary outcomes were safety and well-being in school, mental health status (measured with the Strengths and Difficulties Questionnaire) and educational test scores.

### Estimating costs and resource use

A full economic costing analysis of the Good School Toolkit, as implemented during the trial, was conducted from the perspective of the provider, the NGO Raising Voices. The total costs estimated are related to (1) the development of the Good School Toolkit and (2) start-up and implementation of the Good School Toolkit during the trial.

The development phase (2007–2011) included design, development, pilot and revision of the Toolkit. The start-up phase (September to December 2012) included printing of the Toolkit, introductory workshop and community visits, recruitment and training. The 18-month implementation phase (January 2013 to June 2014) included in-school capacity development and technical support for schools, school-led activities, monitoring and evaluation. The costs related to formative research, baseline survey, randomisation (January to September 2012), end line survey (June to July 2014) and process evaluation were excluded. The costs related to monitoring and evaluation were computed but results are presented with and without them.

Implementation costs were measured over the duration of the trial period during which the intervention package was delivered to 21 intervention schools. Financial and economic costs were collected retrospectively using accounting records, routine monitoring and evaluation data (eg, reports of school-led activities and classroom observations). Interviews with staff of Raising Voices were conducted in August 2014 to assess the percentage of staff time devoted to start-up, implementation and research.

Costs incurred in Ugandan Shillings were converted to US$ using the annual average exchange rate from the Bank of Uganda, and then inflated to 2015 US$ using the International Monetary Fund Consumer Price Index.

The cost of developing the Good School Toolkit was collected as a start-up cost and annualised over the length of the intervention. However, this considerable investment is expected to yield benefits beyond both the duration of the intervention and geographical boundaries of the study setting. Raising Voices staff estimated that the Toolkit would last 7 years before an update would be due. Thus, this cost was treated as a single capital item, annualised over 7 years using a 9.26% discount rate (Ugandan Government Bond yield in 2015) and with resaleable value equal to the original investment. These assumptions were tested in the sensitivity analysis.

Capital costs included office equipment. These were annualised over their expected useful life and discounted at 9.26%. All costs, including staff, overheads (administration, maintenance, management) and capital, were allocated based on proportion of use related to the Good School Toolkit intervention. Costs related to school-led activities were also estimated from monitoring and evaluation reports, as described in the Methods section.

The Good School Toolkit intervention relies on teachers to implement school-led activities, thus in order to inform scalability and sustainability, it is important to estimate the resource time needed to implement these. The opportunity cost of teacher time was also estimated to determine their economic cost and the burden of the intervention on school staff.

The Toolkit has the potential to increase the number of children referred to health and social services, because children may be more likely to report their experiences of violence to school staff. However, we did not document any direct referrals from school staff during the trial as a result of the intervention itself. The additional costs of referral and health cost of treating the consequences of violence are therefore not included. However, in the longer term there is likely to be a cost saving for the social protection and the health system due to number of averted cases of violence. It should be noted that a number of referrals were generated as part of the research process (not the intervention); this is further described elsewhere.^14^

### Outcomes

Unit costs estimated include cost per primary school pupil in intervention school. Cost-effectiveness was estimated as the cost per self-reported case of past-week physical violence averted in primary schools (the main outcome of the trial). The measure of past-week case of physical violence was based on a series of questions on whether the child had experienced different physical acts by a school staff (as described in the main results paper). The number of cases of physical violence averted was estimated as the difference between the number of cases of violence that would have been expected to observe in the intervention schools in the absence of the intervention, and the number of cases actually occurred in intervention schools. The adjusted risk difference in prevalence of past week of violence between intervention and control schools was used to estimate the additional number of cases that would have occurred in intervention schools had the Good School Toolkit not been implemented (expected number).

The adjusted risk difference between intervention and control schools for violence was calculated using a generalised linear model with robust SEs, adjusting for school-level clustering. We adjusted for baseline differences in student and school characteristics and accounting for clustering between student outcomes within schools, as prespecified for intention-to-treat analysis.^10^

95% CIs around the estimates of risk difference were used to calculate upper and lower bounds for the number of cases of violence averted.

### Cost-effectiveness

The cost-effectiveness of the Good School Toolkit intervention was assessed compared with a do-nothing alternative. The cost-effectiveness ratio was calculated as the total cost of implementing the programme divided by the intervention effectiveness (estimated number of cases of physical violence from school staff averted).

### Sensitivity analysis

A number of assumptions had to be made for estimating the costs and cost-effectiveness. To quantify the impact of uncertainty around these assumptions, univariate sensitivity analysis was performed by changing these parameters: (1) design and development costs of the Toolkit were increased/decreased by 50%; (2) ‘resale’ value of the Toolkit at the end of the project was increased/decreased by 50%; (3) number of cases of violence averted using the 95% CI around the intervention-control adjusted risk difference.

### Ethics

The GSS (registered at ClinicalTrials.gov, NCT01678846) included a cluster randomised controlled trial in 42 primary schools in Luwero District, Uganda. The study was approved by the London School of Hygiene and Tropical Medicine Ethics Committee (6183) and the Uganda National Council for Science and Technology (SS2520).

## Results

### Total cost of the Good School Toolkit intervention

The total costs of setting up and running the Good School Toolkit, including process monitor (M&E) activities, are estimated at US$449 845. This includes the total cost of the Good School Toolkit at US$397 233 and total M&E costs at US$52 613. Total costs include set-up and implementation costs relative to staff time, transport and materials. Total staff time (four programme officers, one implementation manager, one peer learning network coordinator and two monitoring officers, plus admin and management support) is estimated at US$211 439, accounting for 47% of total costs. Capital cost (including laptops, office furniture, and so on) is estimated at US$69 164 accounting for 15% of total costs. The remaining 38% includes directly incurred costs such as materials and supplies, transport and utilities for a total of US$169 243. Table 1 reports the results excluding M&E costs.

**Table 1:** Total costs of the Good School Toolkit, excluding M&E costs

	Cost (US$)	%
Total	397 233	
Start-up	117 949	30
Implementation	279 284	70
Staff	156 441	39
Capital	69 164	17
Material, transport, and so on	171 628	43

### Development of the Good School Toolkit and set-up costs

A major component of costs is the initial development costs associated with production of the Toolkit materials. The total estimated cost of designing and developing the Toolkit is US$446 940. Staff time during the period 2007–2011 is estimated at US$244 251 accounting for 55% of the total development costs. Materials used for the development of the Toolkit and financial supports to school during the development phase amount to US$202 689. These costs are a one-off investment, and are treated as capital item with a resaleable value equal to their initial investment. Set-up costs, which are considered repeatable should the intervention be scaled up or rolled out in different areas, include printing of the materials, introductory workshops, recruitment and training of trainers. These costs sum up to US$117 949.

### Implementation costs

The total estimated cost of implementing the Good School Toolkit programme in the trial is US$279 284, excluding M&E, or annual average of US$157 343. The annual cost to run the programme in the trial is US$7493 per school, and US$15 per primary school pupils in the trial intervention schools.

### Cost-effectiveness

The methods and results of the trial are presented in Devries *et al*’s study.^10^ After accounting for clustering between students within schools, the trial results show a 42% reduction in risk of past-week physical violence from school staff.

The number of pupils was estimated at 9000. The expected percentage of cases of violence in intervention schools in the absence of intervention was estimated at 49%, thus the expected number of cases in intervention schools in absence of intervention was estimated at 4410. The observed cases of violence in the intervention area were estimated at 31%, or 2790 cases. The average number of cases averted was calculated as the difference between expected number of cases in intervention schools in absence of intervention and the observed cases in intervention schools.

It is estimated that the intervention has averted 1620 cases of past-week physical violence during the 18-month trial period. Thus, the total cost per case of violence averted is US$245, and the annual implementation cost is US$97 per case averted during the trial.

### Sensitivity analysis

As detailed in tables 2 and 3, the results of univariate sensitivity analysis suggest that the cost and cost-effectiveness estimate are fairly robust in relation to key parameters. The trial costs of setting up and running the Toolkit intervention range from US$332 509 to US$696 808. However, results reveal some uncertainty in relation to effect estimates. The number of cases averted ranges from 2430 to 720, thus the cost per case of violence averted ranges from US$162 to US$548.

**Table 2:** Univariate sensitivity analysis—total costs of the Good School Toolkit (excluding M&E)

Parameter	Lower bound (US$)	Upper bound (US$)
Development costs (±50%)	366 945	435 816
Resale value of Toolkit (±50%)	332 509	696 808

**Table 3:** Univariate sensitivity analysis—costs per case of violence averted

Parameter	Lower bound (US$)	Upper bound (US$)
Development costs (±50%)	227	269
Resale value of Toolkit (±50%)	205	430
Number of cases averted (95% CI 720 to 2430)	162	548

### Teacher time use

The total recorded number of school-led activities is 406 across 21 intervention schools over the implementation period. However, the total number of activities planned was 776 thus the reported number is likely to be an underestimation. The median number of planned activities per school is 38 (IQR: 31–45) and reported 20 (IQR: 15–23). The mean duration of the activities is 1 hour (IQR: 1–2) and the mean number of staff who took part in the activities is 10 (IQR: 7–14).

According to the Service Delivery Indicators initiative, a teacher in Uganda spends 3 hours and 17 min teaching per day (http://www.sdindicators.org/uganda-education/). The school-led activities organised as part of the Good School Toolkit are likely to occur once or twice per month, and are likely to last 1 or 2 hours. Thus, there is no evidence to suggest that the Toolkit is displacing teachers’ time.

The salary of a teacher in a primary school in Uganda is in the range of US$160–US$279 per month, depending on grade and role. The total cost of teacher time to implement the Good School Toolkit activities is estimated between US$1589 and US$2781, across all 21 intervention schools. We estimated an average of US$2185 for the economic cost of teacher time during the implementation of school-based activities.

## Discussion

The Good School Toolkit is the only intervention that has been rigorously evaluated for its ability to reduce staff to student physical violence, and it is highly effective at doing so. During the 18-month implementation period, the Toolkit intervention was delivered in 21 schools, and it averted 1620 cases of physical violence. The cost of running the programme in this period was US$279 284, and the cost of averting one case of physical violence (perpetrated by staff) was US$245. Running costs were US$15 per pupil.

Direct comparison of cost-effectiveness results is not feasible at present since the Good School Toolkit was the first intervention of its kind that has been evaluated via randomised controlled trial, and that has been costed. However, it is useful to compare results with other violence prevention and reduction programmes in the region, even if the outcome measures used and the time period over which benefits are measured are different. SASA! is a programme to prevent IPV against women, and reduce HIV risk. In the SASA! trial, the total estimated economic cost of delivering the intervention was US$582 959 and the estimated cost per case of past-year IPV averted was US$485.^15^ The Intervention with Microfinance for AIDS and Gender Equity trial, which tested the Sisters for Life intervention to prevent intimate partner violence and strengthen gender equity, plus microfinance, in South Africa reports an estimate of US$891 per year free of IPV.^16^ The Good School Toolkit compares favourably against these two interventions.

It is of critical importance to identify sustainable and cost-effective interventions for the prevention of violence against children, especially in resource-poor settings. The Good School Toolkit represents a locally developed, complex behavioural intervention which requires comparatively little time or resources at the school level to deliver. It also does not appear to divert a substantive amount of teacher time, and has the potential to be integrated into the school curriculum.

Running costs were US$15 per pupil during the trial, implying that if a new organisation wanted to implement the Toolkit as it was done during the trial, it could be done for a similar cost, assuming similar levels of competency in staff. If the Toolkit was implemented on a larger scale, there are likely to be some savings made. For example, training can be less expensive if organised through teacher training colleges, and there could also be economies of scale if collaborations are established with other civil society organisations involved already in school improvement initiatives. Raising Voices is currently advocating for uptake of the Toolkit at scale via the Ministry of Education. Further research should accompany this in order to determine the effectiveness and potential cost savings of alternate delivery models.

In addition to reducing physical violence from school staff, it has been demonstrated that the Toolkit also reduced emotional violence and peer violence, and increased students’ connection to the school.^17^ Moreover, the Toolkit changed social norms and attitude on violence and discipline practices, in intervention schools and in the surrounding communities.^18^ Complex behavioural interventions of this nature are likely to generate sustained and positive spillover effects in the future. Estimating these effects in monetary terms, and the number of cases of violence averted in the future, is beyond the scope of the study. However, we acknowledge the fact that tying the economic evaluation to the trial outcome, and the 18-month implementation time frame can be restrictive and can underestimate the true value of the intervention.

Further research is needed on the effectiveness and cost-effectiveness of other school-based violence prevention programmes, particularly now that the Sustainable Development Goals expressly aim to reduce violence against children. Given that most children in most countries attend at least some school, and that many experience violence in this setting,^19^ school-based programmes will be essential in order to achieve these goals. At present, remarkably little information is available on how to prevent school violence, outside of bullying in mainly high-income contexts.^20^ It is also advisable to include in the evaluation design outcome measures that can hold the full breadth of effects of the intervention, for example, measures based on subjective well-being or capabilities.^21^

## Conclusion

The Good School Toolkit is effective in reducing physical violence by teachers against students in Ugandan primary schools. Costs compare favourably with other violence prevention interventions in the region, but no direct comparators exist. Further research on the effectiveness and cost-effectiveness of other similar interventions which could also incorporate broader outcome measures is urgently needed.
